# Characterization of *PR-Cre* Activity in the Testis and Its Application Reveals BRG1 Is Dispensable in Adult Leydig Cells

**DOI:** 10.3390/biom16060816

**Published:** 2026-05-31

**Authors:** Hongbiao Shi, Yilin Du, Yu Liang, Ai Liu, Congzhe Hou, Xi Li, Jiangxia Li, Wenjie Sun, Yecheng Jin, Qiji Liu

**Affiliations:** 1 Key Laboratory for Experimental Teratology of the Ministry of Education, Department of Medical Genetics, School of Basic Medical Sciences, Cheeloo College of Medicine, Shandong University, Jinan 250012, China; 2 School of Life Science, Shandong University, Jinan 250100, China; 3 Experimental Center, Shandong University of Traditional Chinese Medicine, Jinan 250355, China; 4 NHC Key Laboratory of Cardiopulmonary Rehabilitation and Functional Recovery, School of Health and Life Sciences, University of Health and Rehabilitation Sciences, Qingdao 266113, China

**Keywords:** Leydig cells, BRG1, *PR-Cre*, testes, male fertility

## Abstract

Leydig cells play a crucial role in male development, fertility, and overall health through hormone production. Brahma-related gene 1 (BRG1), the catalytic subunit of the mammalian SWI/SNF complex, is a key regulator of chromatin accessibility and governs the development and function of diverse tissues. However, its role in Leydig cells remains unclear. In this study, we first characterized the expression pattern of *PR-Cre* in the testes, as this Cre mouse line has been widely used for gene targeting in the female reproductive system, but its activity in the testis has never been systematically reported. We found that *PR-Cre* drives recombination in multiple testicular cell types, including stem/progenitor adult Leydig cells (ALCs), peritubular myoid cells, and elongated spermatids. Using *PR-Cre* to conditionally delete BRG1 in ALCs, we observed no detectable abnormalities in ALC development, spermatogenesis, or male fertility. Similar results were obtained using the *Cyp17a1-iCre* mouse line and AAV8-iCre viral delivery for BRG1 deletion. Collectively, this work demonstrates that BRG1 is dispensable for ALC development and function, while providing a comprehensive characterization of *PR-Cre* as a valuable new tool for male reproductive research.

## 1. Introduction

Testicular Leydig cells serve as the primary source of androgens in males, playing indispensable roles in sexual differentiation, spermatogenesis, and the maintenance of secondary sexual characteristics [1,2]. In the mouse testis, Leydig cells exist in two distinct populations: fetal Leydig cells (FLCs), which arise during embryonic development and are responsible for masculinization of the male reproductive tract, and adult Leydig cells (ALCs), which emerge after birth and produce the high levels of testosterone required for spermatogenesis and fertility [3,4,5]. The ALC population differentiates from stem/progenitor cells during the postnatal period, ultimately becoming the sole testicular source of de novo testosterone synthesis [3,4]. The steroidogenic function of Leydig cells is governed by a precisely orchestrated transcriptional network, with the orphan nuclear receptor steroidogenic factor 1 (SF-1, NR5A1) serving as a master regulator that controls the expression of key steroidogenic enzymes, including STAR, CYP17A1, and HSD3B1 [6,7]. In recent years, increasing attention has been directed toward the role of chromatin remodeling in steroidogenic gene regulation [8,9,10].

ATP-dependent chromatin remodeling complexes are major epigenetic regulators that use ATP hydrolysis to modulate nucleosome structure, thereby facilitating gene activation or repression [11]. A key member of these complexes is the mammalian SWItch/Sucrose Non-Fermentable (mSWI/SNF) complex, also known as the BRG1/BRM-associated factor (BAF) complex [12]. BRG1 (Brahma-related gene 1), the core catalytic ATPase subunit of the BAF complex, is essential for its chromatin remodeling activity. Through ATP hydrolysis, the BAF complex remodels nucleosome positioning to modulate chromatin accessibility and gene transcription [12]. Accumulating evidence shows that BRG1 is critical for proper development and function across multiple tissues and organs, and its dysregulation is linked to various pathologies [13,14]. Consistent with this, our previous studies have demonstrated essential roles for BRG1 in the development of the inner ear [15,16,17] and cerebral cortex [18]. In the testis, BRG1 is dynamically expressed in spermatogenic cells and plays an important role during spermatogenesis. Specific deletion of BRG1 in the germline leads to defects in spermatogenesis and male infertility, primarily due to impaired DNA repair and abnormal chromatin modifications [19,20]. In contrast, Sertoli cell development and function are not affected by loss of BRG1 [21]. However, the function of BRG1 in Leydig cells remains unclear.

To investigate the cell-autonomous role of BRG1 in Leydig cells, the use of Cre-loxP technology is essential, and the selection of appropriate Cre driver lines is critical. The *Cyp17a1-iCre* line has been widely used to target Leydig cells, as *Cyp17a1* is a well-established marker of steroidogenic cells [22,23,24,25]. In parallel, the *PR-Cre* (*progesterone receptor-Cre*, also known as *Pgr-Cre*) line was originally generated for studying progesterone receptor-expressing cells in the female reproductive tract and has been extensively characterized in the uterus, ovary, and oviduct [26]. Importantly, the progesterone receptor itself has been detected in the testis, with expression reported in Leydig cells, Sertoli cells, and sperm cells [27,28,29], raising the possibility that *PR-Cre* may also exhibit activity in the male gonad. Notably, however, the expression pattern and recombination efficiency of *PR-Cre* in the male testis have never been systematically reported. Consistent with this, a recent comprehensive review of Cre driver lines for male reproductive research did not include *PR-Cre* in its inventory [30]. This represents a significant knowledge gap, as *PR-Cre* could potentially serve as an alternative tool for targeting testicular cell populations if its expression in the male gonad is characterized.

In this study, we sought to address two interrelated questions. First, we aimed to determine whether BRG1 is required for Leydig cell development and function in vivo by conditionally ablating it using the Cre-loxP system. Second, we took the opportunity to systematically characterize the expression pattern of *PR-Cre* in the testis. Unexpectedly, mice with ALC-specific deletion of BRG1 exhibited normal testicular morphology, spermatogenesis, and fertility. More importantly, our characterization of *PR-Cre* revealed robust Cre activity in testicular stem/progenitor ALCs, peritubular myoid cells, and elongated spermatids, establishing this line as a valuable new tool for gene targeting in the testis that has been overlooked in the field. Collectively, this work not only demonstrates that BRG1 is dispensable for ALC development and function but also provides a novel methodological resource by validating *PR-Cre* as a previously unrecognized driver for gene ablation in testicular cells.

## 2. Materials and Methods

### 2.1. Mice

All animal experimental procedures were approved by the Ethics Committee of the School of Basic Medical Sciences, Shandong University (approval No. ECSBMSSDU2024-2-29; approval date: 29 February 2024). The animals were housed under standard conditions at 22 ± 1 °C with a 12 h light/dark cycle and were provided free access to food and water throughout the study. *Brg1^flox^*^/*flox*^ [31], *PR-Cre* [32], *Cyp17a1-iCre* [22] and *Rosa26-tdTomato* [33] mouse lines were maintained on a mixed genetic background and genotyped as previously described [22,31,32,33].

For timed pregnancies, the day of vaginal plug detection was regarded as E0.5 and the day of birth as P0.

### 2.2. Immunofluorescence Staining

Testes were dissected and fixed in Bouin’s solution or 4% paraformaldehyde (PFA) in phosphate-buffered saline (PBS) at 4 °C overnight. Fixed testes were dehydrated through a graded ethanol series (30%, 50%, 70%, 15 min each; 80%, 95%, 30 min each; absolute ethanol I and II, 50 min each), cleared in ethanol:xylene mixtures (1:1 and 1:2, 20 min each) followed by pure xylene (≤10 min), infiltrated with xylene:paraffin mixtures (2:1 and 1:1, 20 min each) and three changes in pure paraffin (30, 45, and 60 min), then embedded and sectioned at 5 µm thickness. Testis sections went through antigen retrieval by boiling in 10 mM sodium citrate, pH 6.0, for 20 min, cooling naturally to room temperature. Samples were blocked for 30 min with 10% donkey serum, followed by incubation with primary antibodies in PBS at 4 °C overnight. After three washes with PBS, samples were incubated at 37 °C for 1 h in secondary antibodies. 4′,6-diamidino-2-phenylindole (DAPI) was used to stain the nuclei. Images were acquired using an OLYMPUS BX53 microscope (Olympus Corporation, Tokyo, Japan).

The following primary antibodies were used for immunostaining: anti-DDX4 (Rabbit, 1:400, ab270534, abcam, Cambridge, UK), anti-CYP17A1 (Rabbit, 1:400, 14447-1-AP, Proteintech, Wuhan, China), anti-αSMA (Mouse, 1:400, GB13044, Servicebio, Wuhan, China), anti-Nestin (Rabbit, 1:400, 89529, CST, Danvers, MA, USA), anti-BRG1 (Rabbit, 1:400, ab110641, abcam, Cambridge, UK), anti-γ-H2AX (Rabbit, 1:400, 9718, CST, Danvers, MA, USA).

### 2.3. Male Fertility Test

One male mouse (6–12 weeks old) was continuously housed with two WT C57BL/6 females (6–12 weeks old). The females were checked for vaginal plugs every morning. Upon detection of a plug, the plugged female was replaced with a naive female to continue mating. The plugged female was then separated, housed singly, and monitored for pregnancy. After delivery, the litter size was recorded. This fertility test lasted for 4 months. At least two litters were obtained per male, and the average litter size was calculated for each male as the final value for that animal.

### 2.4. Computer-Assisted Sperm Analysis (CASA)

The epididymis was dissected and placed into a microcentrifuge tube containing 1 mL of pre-warmed human tubal fluid (HTF, 37 °C) and minced thoroughly using sterile scissors. The sample was then incubated at 37 °C in a metal bath for 15 min to allow spermatozoa to swim out. A 10 μL aliquot of the sperm suspension from the cauda epididymis was used to assess sperm motility and velocity using a CASA system (TOX IVOS-II, Hamilton Thorne, Hamilton Thorne, Inc., Beverly, MA, USA). Six mice per group (control and experimental) were analyzed. At least four microscopic fields were analyzed per mouse, and the mean values were used for all reported motility parameters. The evaluated parameters included: percentage of motile sperm, percentage of progressive motility, percentage of rapid progressive motility, average path velocity (VAP), straight-line velocity (VSL), and curvilinear velocity (VCL).

### 2.5. Measurement of Serum Testosterone Hormone Levels

Blood was collected from mice via retro-orbital bleeding and allowed to clot at room temperature for 30–60 min. Serum was then separated by centrifugation at 3000× *g* for 10 min at 4 °C. Serum testosterone levels were measured using a competitive enzyme-linked immunosorbent assay (ELISA) kit (PT872, Biotech Inc., Shanghai, China) according to the manufacturer‘s instructions. Serum samples from four mice per group (control and experimental) were analyzed. For each mouse, three technical replicate wells were measured, and the average value was calculated as the value for that mouse.

### 2.6. Reverse Transcription Quantitative Polymerase Chain Reaction (RT-qPCR)

Testes were dissected, and total RNA was extracted using RNAiso Plus reagent (Cat# 9109, Takara Bio Inc., Kusatsu, Shiga, Japan) according to the manufacturer’s instructions. cDNA was synthesized using HiScript III RT SuperMix for qPCR (with gDNA wiper) (R323-01, Nanjing Vazyme Biotech Co., Ltd., Nanjing, China). Real-time quantitative PCR was performed using SuperStar Universal SYBR Master Mix (CW3360H, Jiangsu Cowin Biotech Co., Ltd., Taizhou, China) with gene-specific primers (Supplementary Materials, Table S1). The 2^−ΔΔCt^ method was used to calculate relative gene expression levels. For each mouse, four technical replicate wells were analyzed, and the average value was calculated as the expression level for that mouse.

### 2.7. AAV8-iCre Delivery in Animal Models

The interstitial injection of PBS or AAV8-iCre vectors into mouse testes was performed based on a previously described protocol [34], with some modifications. Briefly, *Brg1^flox/flox^* or *Rosa26-tdTomato* mice were anesthetized with pentobarbital sodium (50 mg/kg) by intraperitoneal injection. The surgical site was sterilized with ethanol followed by topical application of povidone-iodine. A single incision was made on the ventral skin and body wall approximately 1.5 cm anterior to the genitals using sterile surgical scissors under aseptic conditions. The testes were gently exteriorized by holding the fat pad, taking care not to injure the blood vessels. Each testis was immobilized with fine forceps, and 10 μL of either AAV8-iCre (Shanghai GeneChem Co., Ltd., Shanghai, China) (at a titer of 8 × 10^10^ viral genomes per mL) or PBS, pre-measured with a calibrated micropipette, was drawn into a BD Insulin syringe and injected into the interstitial space. The incision was then sutured.

### 2.8. Statistics

All quantitative data were analyzed using GraphPad Prism 5 software (La Jolla, CA, USA). Normality of the data was assessed using the Shapiro–Wilk test. For comparisons between two groups, an unpaired two-tailed Student’s *t*-test was applied when the data were normally distributed. Results are presented as mean ± SD. Sample sizes (*n*) representing the number of animals per group are indicated in the figure legends.

## 3. Results

### 3.1. Expression of PR-Cre in the Testis

To assess the expression pattern of *PR-Cre*, we crossed *PR-Cre* mice [32] with *Rosa26-tdTomato* reporter mice, in which tdTomato expression is activated upon Cre-mediated excision of a loxP-flanked transcriptional STOP cassette [33]. Mice carrying one copy of the *PR-Cre* allele and one copy of the *Rosa26-tdTomato* allele (hereafter referred to as *PR-tdTomato*) were analyzed at various developmental stages, with *Rosa26-tdTomato* single-transgenic littermates serving as controls.

**Figure 1:**
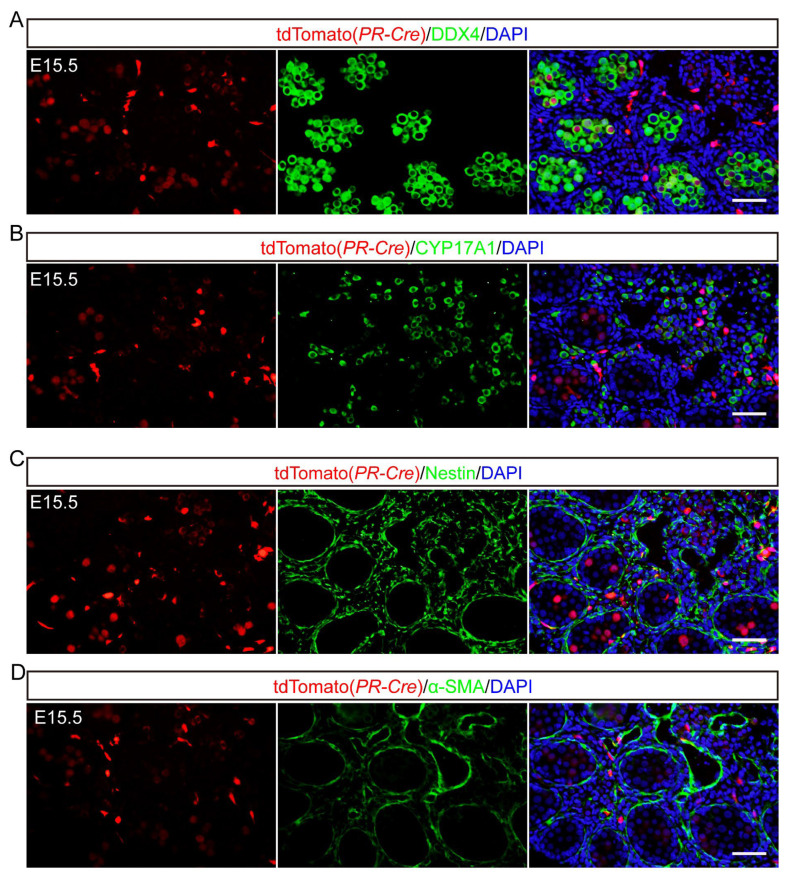
*PR-Cre*-mediated recombination pattern in embryonic testes at E15.5. Cryosections of E15.5 testes from *PR-tdTomato* mice were immunostained for DDX4 (**A**), CYP17A1 (**B**), Nestin (**C**), and α-SMA (**D**). tdTomato fluorescence indicates Cre-mediated recombination. Nuclei were counterstained with DAPI. Scale bars: 50 μm.

At embryonic stages, tdTomato-positive cells were sparse, indicating very limited Cre activity. Within the testis cords, Cre-mediated recombination was detected only in a small subset of DDX4-positive germ cells (Figure 1A). In the interstitium, no tdTomato signal was detected in CYP17A1-positive FLCs (Figure 1B). Scattered tdTomato-positive cells were occasionally observed, most of which were Nestin-positive and closely associated with α-SMA staining, although the tdTomato-positive cells themselves were negative for α-SMA (Figure 1C,D).

**Figure 2:**
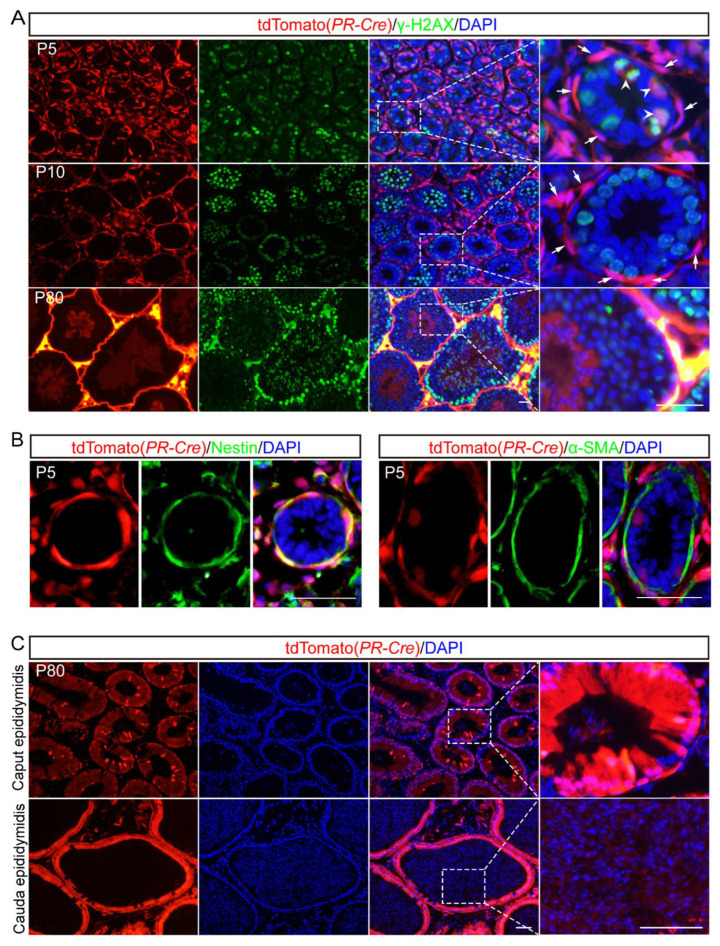
*PR-Cre*-mediated recombination pattern in postnatal testes and epididymis. (**A**) Cryosections of *PR-tdTomato* testes at P5, P10, and P80 were immunostained for γ-H2AX. Arrowheads indicate tdTomato-positive spermatogonia; arrows indicate tdTomato-positive stem/progenitor ALCs. (**B**) Cryosections of *PR-tdTomato* testes at P5 were immunostained for Nestin and α-SMA. (**C**) Cryosections of *PR-tdTomato* epididymis at P80. tdTomato fluorescence indicates Cre-mediated recombination. Nuclei were counterstained with DAPI. Scale bars: 50 μm.

During postnatal development, tdTomato expression was detected in a small subset of γ-H2AX-positive germ cells within the seminiferous tubules (at P5: 18.11% ± 1.34% of γ-H2AX-positive cells were tdTomato-positive; at P10: 13.67% ± 1.80%) (Figure 2A), similar to the pattern observed during embryogenesis. In the interstitium, robust tdTomato expression was observed in a population of spindle-shaped peritubular cells surrounding the seminiferous tubules (Figure 2A). Immunostaining with Nestin (a stem/progenitor ALC marker) and α-SMA (a peritubular myoid cell marker) confirmed that these tdTomato-positive spindle-shaped cells were Nestin-positive but α-SMA-negative (at P5: 87.37% ± 1.43% of Nestin-positive cells were tdTomato-positive), indicating that they are stem/progenitor ALCs rather than peritubular myoid cells (Figure 2B). In adult mice, tdTomato expression was detected in nearly all peritubular myoid cells and ALCs (at P80: 94.40% ± 1.65% of interstitial cells were tdTomato-positive), as well as in all elongated spermatids and the resulting spermatozoa (Figure 2A,C). In contrast, tdTomato expression was largely undetectable in spermatogonia and meiotic spermatocytes at earlier stages (Figure 2A). This expression pattern indicates that *PR-Cre* exhibits stage-specific activity during spermatogenesis, with particularly robust recombination occurring in elongated spermatids, rather than reflecting persistent tdTomato protein from earlier recombination events. Beyond the testis, *PR-Cre* activity was also detected in the epididymis, where tdTomato expression was observed in most principal cells throughout the epididymis (caput: 85.63% ± 4.00% principal cells were tdTomato-positive; cauda: 90.33% ± 2.67%), as well as in peritubular smooth muscle cells lining the cauda epididymis (Figure 2C).

Collectively, these findings demonstrate that *PR-Cre* exhibits minimal and sporadic activity during embryogenesis, with robust Cre activation occurring postnatally. Postnatal *PR-Cre* targets multiple testicular cell types, including stage-specific germ cells, peritubular myoid cells, and stem/progenitor ALCs, positioning it as a valuable tool for studying late spermatogenesis and testicular somatic cell function.

### 3.2. Expression of BRG1 in the Testis and Epididymis

**Figure 3:**
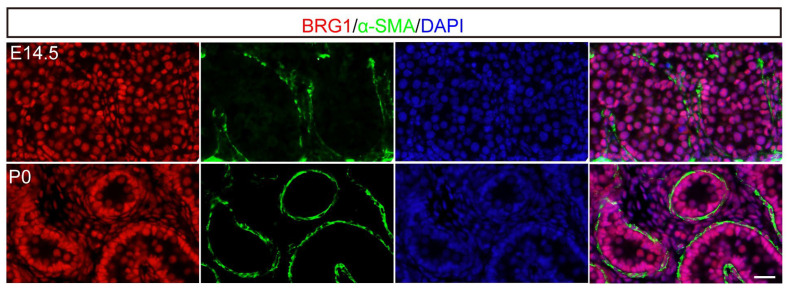
BRG1 expression in embryonic and neonatal testes. Sections of mouse testes at E14.5 and P0.5 were immunostained for BRG1 and α-SMA. Nuclei were counterstained with DAPI. Scale bar: 25 μm.

**Figure 4:**
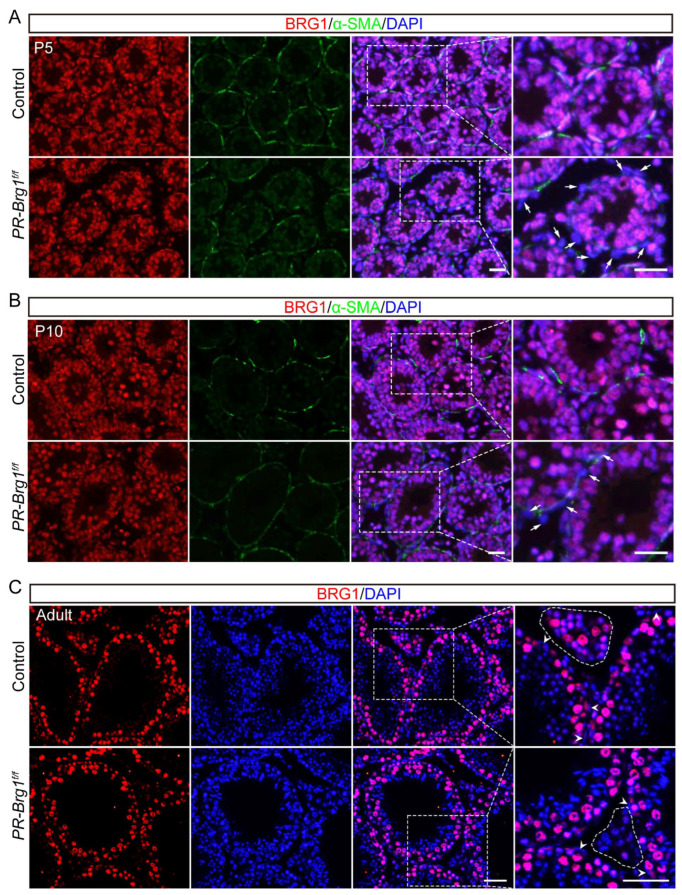
BRG1 deletion in *PR-Brg1^f/f^* testes. (**A**) Sections of control and *PR-Brg1^f/f^* testes at P5 were immunostained for BRG1 and α-SMA. Arrows indicate stem/progenitor ALCs with reduced BRG1 signal compared to controls. (**B**) Sections of control and *PR-Brg1^f/f^* testes at P10 were immunostained for BRG1 and α-SMA. Arrows indicate stem/progenitor ALCs with undetectable BRG1 signal. (**C**) Sections of adult control and *PR-Brg1^f/f^* testes were immunostained for BRG1. In the rightmost panel, the dashed line outlines interstitial cells (mainly Leydig cells). Arrowheads indicate peritubular myoid cells, which are BRG1-negative in both control and *PR-Brg1^f/f^* testes. Nuclei were counterstained with DAPI. Scale bar: 50 μm.

To investigate the potential role of BRG1 in Leydig cells, we first examined its expression pattern at different developmental stages by immunofluorescence. At embryonic day (E) 14.5, BRG1 was ubiquitously expressed in all cell types within the testis, with comparable signal intensities across different cell lineages (Figure 3). During postnatal testicular development, BRG1 was continuously detected in cells within the interstitium. Notably, its expression level in ALCs was relatively lower than that observed at earlier stages (Figure 3 and Figure 4). Within the germ cell lineage, BRG1 was strongly expressed in spermatogonia and spermatocytes, with its expression gradually decreasing as spermatids matured; elongated spermatids showed little to no BRG1 signal (Figure 4), consistent with previous reports [19,35]. In Sertoli cells, BRG1 expression persisted throughout development (Figure 4), also consistent with previous findings [21]. In contrast, BRG1 expression in peritubular myoid cells progressively declined as the testis matured, becoming virtually undetectable in adult peritubular myoid cells (Figure 4).

**Figure 5:**
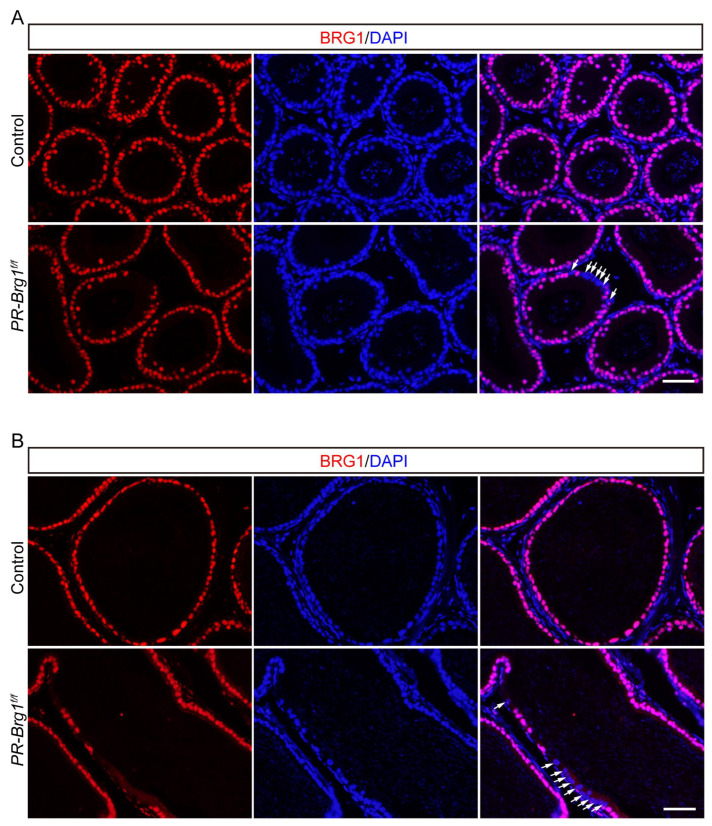
BRG1 deletion in *PR-Brg1^f/f^* epididymis. (**A**) Sections of adult control and *PR-Brg1^f/f^* caput epididymidis were immunostained for BRG1. (**B**) Sections of adult control and *PR-Brg1^f/f^* cauda epididymidis were immunostained for BRG1. Arrows indicate principal cells with undetectable BRG1 signal. Nuclei were counterstained with DAPI. Scale bars: 50 μm.

We also assessed BRG1 expression in the adult epididymis. Immunofluorescence revealed strong BRG1 expression in basal cells surrounding the epididymal lumen, whereas spermatozoa within the lumen were negative (Figure 5). Other cell types in the epididymal epithelium exhibited negligible BRG1 signal (Figure 5).

### 3.3. Deletion of BRG1 in ALCs Using PR-Cre

To investigate the function of BRG1 in Leydig cells, we crossed *Brg1^flox/flox^* mice with *PR-Cre* mice to generate a *PR-Cre*-driven Brg1 knockout mouse model (*PR-Brg1^f/f^* mice). We first assessed the efficiency of BRG1 deletion in target cells. At postnatal day (P) 5 and P10, efficient BRG1 ablation was observed in stem/progenitor ALCs (Figure 4A,B). In adult *PR-Brg1^f/f^* mice, BRG1 was deleted in nearly all ALCs (93.81% ± 1.35% of interstitial cells were BRG1-negative) (Figure 4C).

Because *PR-Cre* also exhibits activity in the epididymis, we further examined BRG1 deletion in this tissue. In the epididymis, BRG1 ablation was detected in only a minor fraction of principal cells (caput: 2.93% ± 0.92% of principal cells were BRG1-negative; cauda: 5.97% ± 3.65%) (Figure 5), which differed from the tdTomato reporter activity, where tdTomato expression was observed in a broader subset of principal cells (Figure 2B). This discrepancy may be attributed to the higher recombination threshold required for biallelic *Brg1* excision compared with single-allele reporter activation, as well as potential differences in chromatin accessibility between the *Rosa26* and *Brg1* loci. Alternatively, lower Cre expression levels in some principal cells may suffice to activate the reporter but not to achieve complete BRG1 deletion.

Together, these results demonstrate that *PR-Cre* efficiently drives BRG1 deletion in ALCs and their progenitors, establishing a suitable model for studying BRG1 function in this cell population.

### 3.4. PR-Cre-Mediated BRG1 Deletion in ALCs Does Not Affect Male Fertility

**Figure 6:**
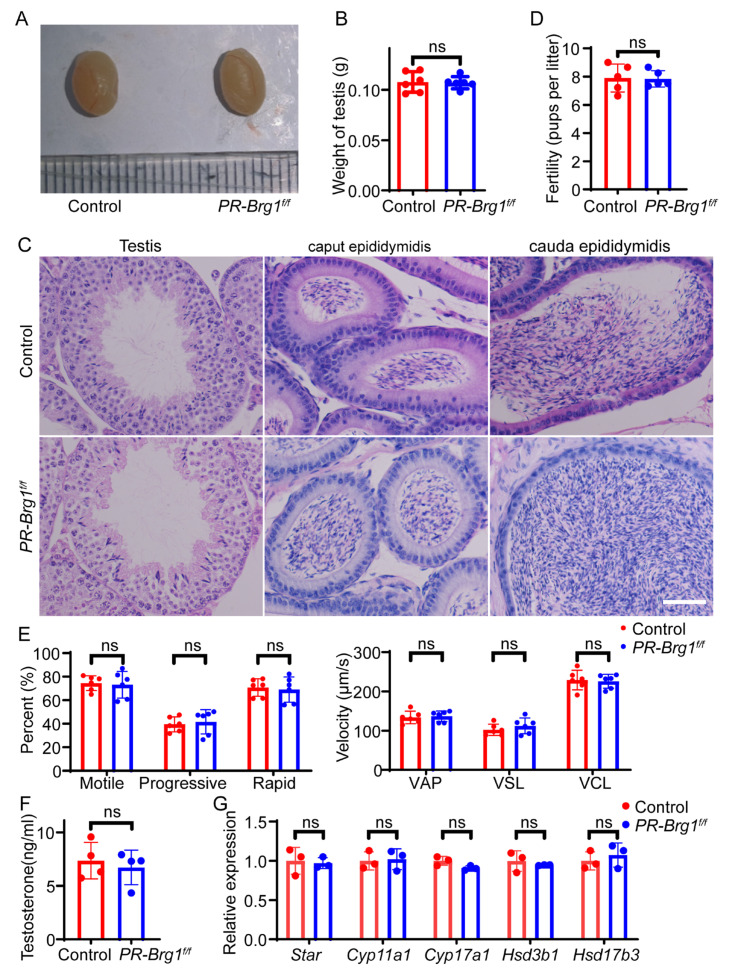
*PR-Brg1^f/f^* male mice exhibit normal testicular morphology and fertility. (**A**) Representative images of testes from adult control and *PR-Brg1^f/f^* mice show comparable size. (**B**) Quantification of testis weight in adult control and *PR-Brg1^f/f^* mice. *n* = 6 for each group. (**C**) H&E-stained sections of adult control and *PR-Brg1^f/f^* testes and epididymis. (**D**) Quantification of the number of pups per litter. *n* = 5 for each group. (**E**) Sperm motility parameters assessed by CASA, including motile sperm (%), progressive motility (%), rapid progressive motility (%), average path velocity (VAP, μm/s), straight-line velocity (VSL, μm/s), and curvilinear velocity (VCL, μm/s). *n* = 6 for each group. (**F**) Serum testosterone levels measured by ELISA in control and *PR-Brg1^f/f^* mice. *n* = 4 for each group. (**G**) RT-qPCR analysis of steroidogenic gene expression (*Star*, *Cyp11a1*, *Cyp17a1*, *Hsd3b1*, *and Hsd17b3*) in control and *PR-Brg1^f/f^* testes. *n* = 3 for each group. Error bars represent SD. ns, not significant, Student’s *t*-tests. Scale bar: 50 μm.

To assess the functional consequences of BRG1 ablation in ALCs, we analyzed testes from control and *PR-Brg1^f/f^* littermates. No significant differences were observed in gross appearance or weight of the testes between the two groups (Figure 6A,B). Histological examination by Hematoxylin and eosin (H&E) staining revealed normal ALC morphology in *PR-Brg1^f/f^* mice (Figure 6C). Moreover, all stages of spermatogenic cells were present within the seminiferous tubules, with elongated spermatids observed in the adluminal compartment (Figure 6C). The presence of mature sperm in the caput and cauda epididymidis was also comparable to controls (Figure 6C). To evaluate whether BRG1 deletion in ALCs affects male fertility, we performed breeding assays. The average litter size from mating pairs involving *PR-Brg1^f/f^* males was not significantly different from that of control littermates (Figure 6D). To further assess ALC function, we measured sperm motility and serum testosterone levels, and no significant differences were detected between control and *PR-Brg1^f/f^* mice (Figure 6E,F). In addition, RT-qPCR analysis of key steroidogenic genes—including Star, Cyp11a1, Cyp17a1, Hsd3b1, and Hsd17b3—revealed no significant differences in expression levels between the two groups (Figure 6G).

Collectively, these results demonstrate that conditional deletion of BRG1 in ALCs driven by *PR-Cre* does not impair ALC development or function, and has no detectable impact on spermatogenesis or male fertility.

### 3.5. Cyp17a1-iCre-Mediated BRG1 Deletion in ALCs Does Not Affect Male Fertility

**Figure 7:**
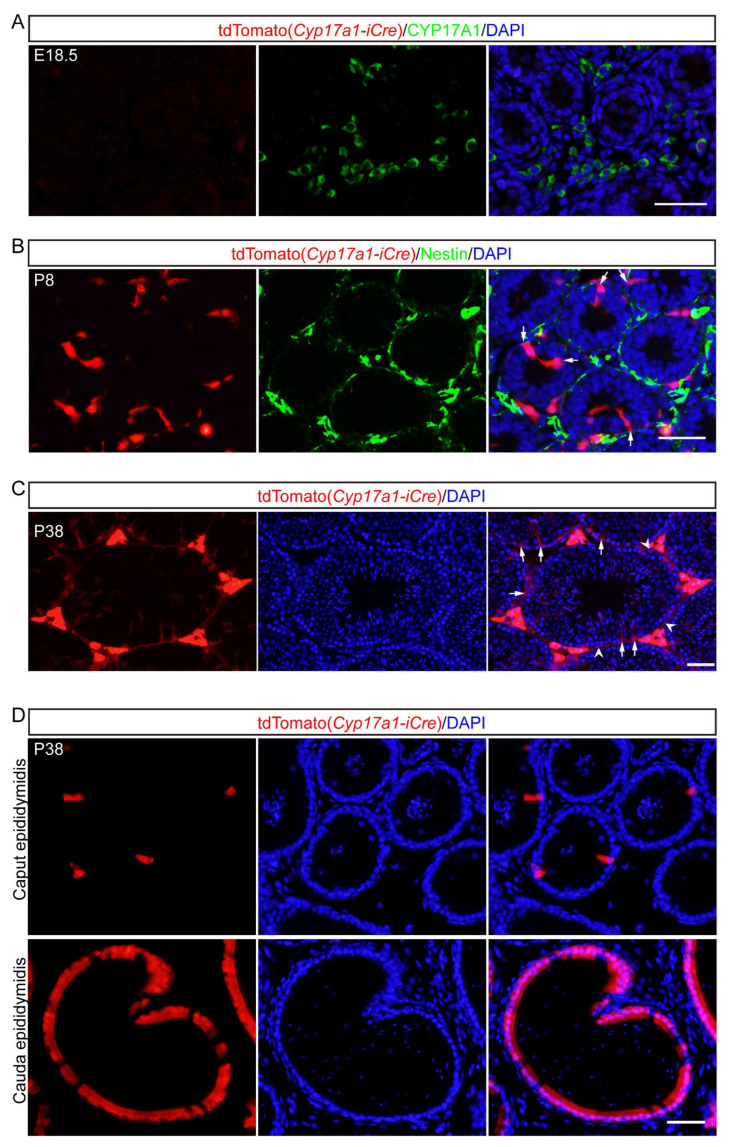
*Cyp17a1-iCre*-mediated recombination pattern in testes and epididymis. (**A**) Cryosections of *Cyp17a1-tdTomato* testes at E18.5 were immunostained for CYP17A1. (**B**) Cryosections of *Cyp17a1-tdTomato* testes at P8 were immunostained for Nestin. (**C**) Cryosections of *Cyp17a1-tdTomato* testes at P38. (**D**) Cryosections of *Cyp17a1-tdTomato* epididymis at P30. tdTomato fluorescence indicates Cre-mediated recombination. Nuclei were counterstained with DAPI. Arrowheads indicate tdTomato-positive peritubular myoid cells; arrows indicate tdTomato-positive Sertoli cells. Scale bars: 50 μm.

To independently validate the dispensability of BRG1 in ALCs, we used the *Cyp17a1-iCre* mouse line—a well-established model for targeted gene deletion in Leydig cells [22,23,24,25]—to conditionally delete BRG1. We first crossed these mice with *Rosa26-tdTomato* reporter mice to characterize the spatiotemporal expression pattern of *Cyp17a1-iCre* in the testis. During embryonic development, we detected little to no Cre activity in the testis (Figure 7A). This observation differs from a previous report showing strong *Cyp17a1-iCre* activity in fetal testis [22], a discrepancy that may reflect differences in reporter sensitivity or genetic background. At P8, sporadic tdTomato-positive cells were observed in the interstitium, with morphology consistent with differentiated ALCs (Figure 7B). By P38, abundant tdTomato-positive ALCs were detected (88.15% ± 1.84% of interstitial cells were tdTomato-positive) (Figure 7C). In addition to ALCs, we also observed tdTomato expression in a small subset of Sertoli cells and peritubular myoid cells (Figure 7B,C), an observation that has not been previously reported for this Cre line. These results indicate that *Cyp17a1-iCre* predominantly drives recombination in differentiated ALCs, albeit with occasional ectopic activity in other somatic cell types.

We also assessed *Cyp17a1-iCre* activity in the epididymis and observed tdTomato expression in principal cells (Figure 7D). The uniformity of tdTomato expression varied across epididymal regions: principal cells in the cauda showed the strongest signal, while only a small proportion in the caput were tdTomato-positive (caput: 3.59% ± 0.37%; cauda: 91.24% ± 2.03%) (Figure 7D), consistent with earlier reports [36].

**Figure 8:**
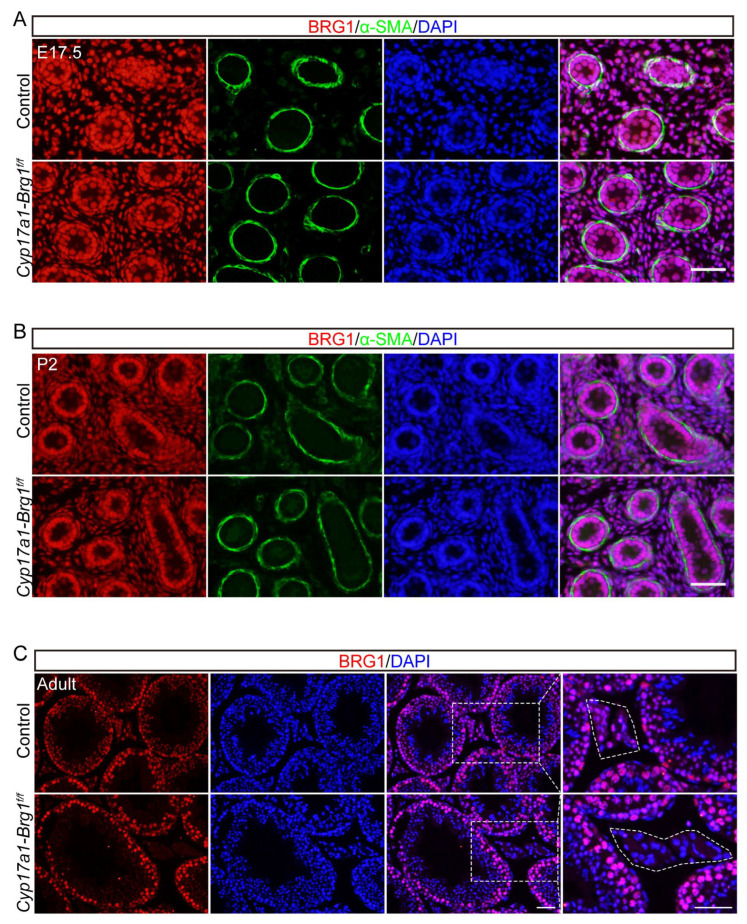
BRG1 deletion in *Cyp17a1-Brg1^f/f^* testes. (**A**) Sections of control and *Cyp17a1-Brg1^f/f^* testes at E17.5 were immunostained for BRG1 and α-SMA. (**B**) Sections of control and *Cyp17a1-Brg1^f/f^* testes at P1.5 were immunostained for BRG1 and α-SMA. (**C**) Sections of adult control and *Cyp17a1-Brg1^f/f^* testes were immunostained for BRG1. In the rightmost panel, the dashed line outlines interstitial cells (mainly Leydig cells). Nuclei were counterstained with DAPI. Scale bars: 50 μm.

**Figure 9:**
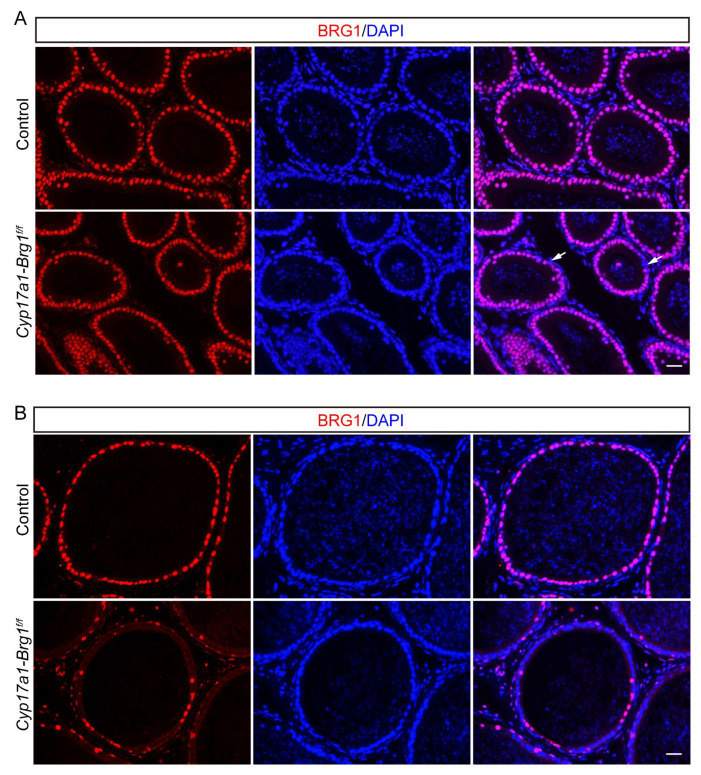
BRG1 deletion in *Cyp17a1-Brg1^f/f^* epididymis. (**A**) Sections of adult control and *Cyp17a1-Brg1^f/f^* caput epididymidis were immunostained for BRG1. Arrows indicate principal cells with undetectable BRG1 signal. (**B**) Sections of adult control and *Cyp17a1-Brg1^f/f^* cauda epididymidis were immunostained for BRG1. Nuclei were counterstained with DAPI. Scale bars: 50 μm.

We next generated ALC-specific BRG1 knockout mice (*Cyp17a1-Brg1^f/f^* mice) by crossing *Brg1^f/f^* mice with *Cyp17a1-iCre* mice. Immunofluorescence analysis revealed no detectable BRG1 deletion in the testes of embryonic or neonatal mutant mice (Figure 8A,B). In adult mutants, however, efficient BRG1 deletion was confirmed in testicular ALCs (93.96% ± 0.97% of interstitial cells were BRG1-negative) (Figure 8C). Deletion was also observed in most principal cells in the cauda epididymis, while principal cells in the caput retained largely normal BRG1 expression (caput: 2.16% ± 0.96% principal cells were BRG1-negative; cauda: 88.00% ± 0.35%) (Figure 9), correlating with the regional activity pattern of *Cyp17a1-iCre*.

**Figure 10:**
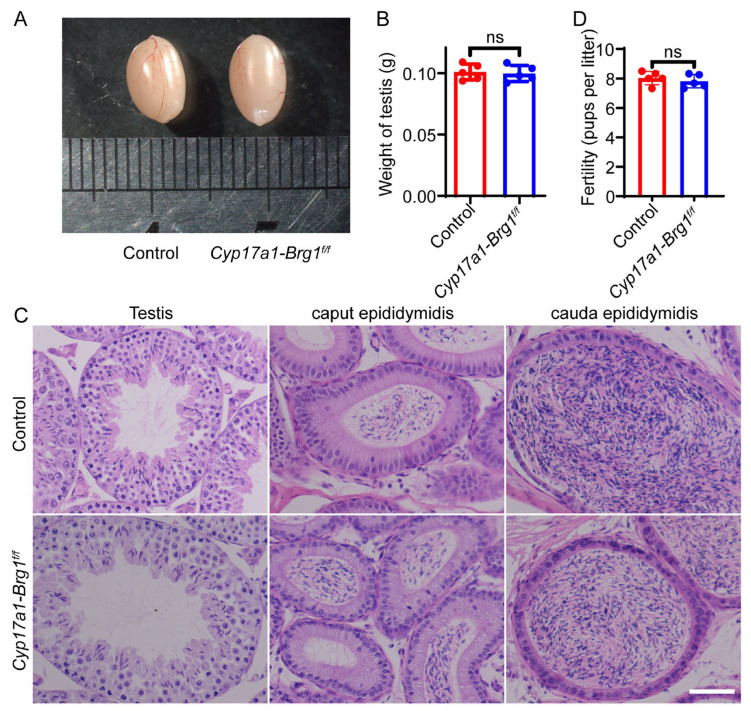
*Cyp17a1-Brg1^f/f^* male mice exhibit normal testicular morphology and fertility. (**A**) Representative images of testes from adult control and *Cyp17a1-Brg1^f/f^* mice show comparable size. (**B**) Quantification of testis weight in adult control and *Cyp17a1-Brg1^f/f^* mice. *n* = 5 for each group. (**C**) H&E-stained sections of adult control and *Cyp17a1-Brg1^f/f^* testes and epididymis. (**D**) Quantification of the number of pups per litter. *n* = 5 for each group. Error bars represent SD. ns, not significant, Student’s *t*-tests. Scale bar: 50 μm.

Consistent with the findings from *PR-Cre*-mediated knockout, ablation of BRG1 using the *Cyp17a1-iCre* driver likewise resulted in no discernible phenotypic alterations. Mutant mice exhibited normal testicular morphology and histology, complete spermatogenesis, and unaltered male fertility (Figure 10).

### 3.6. AAV8-iCre-Mediated BRG1 Deletion in ALCs Does Not Affect Male Fertility

**Figure 11:**
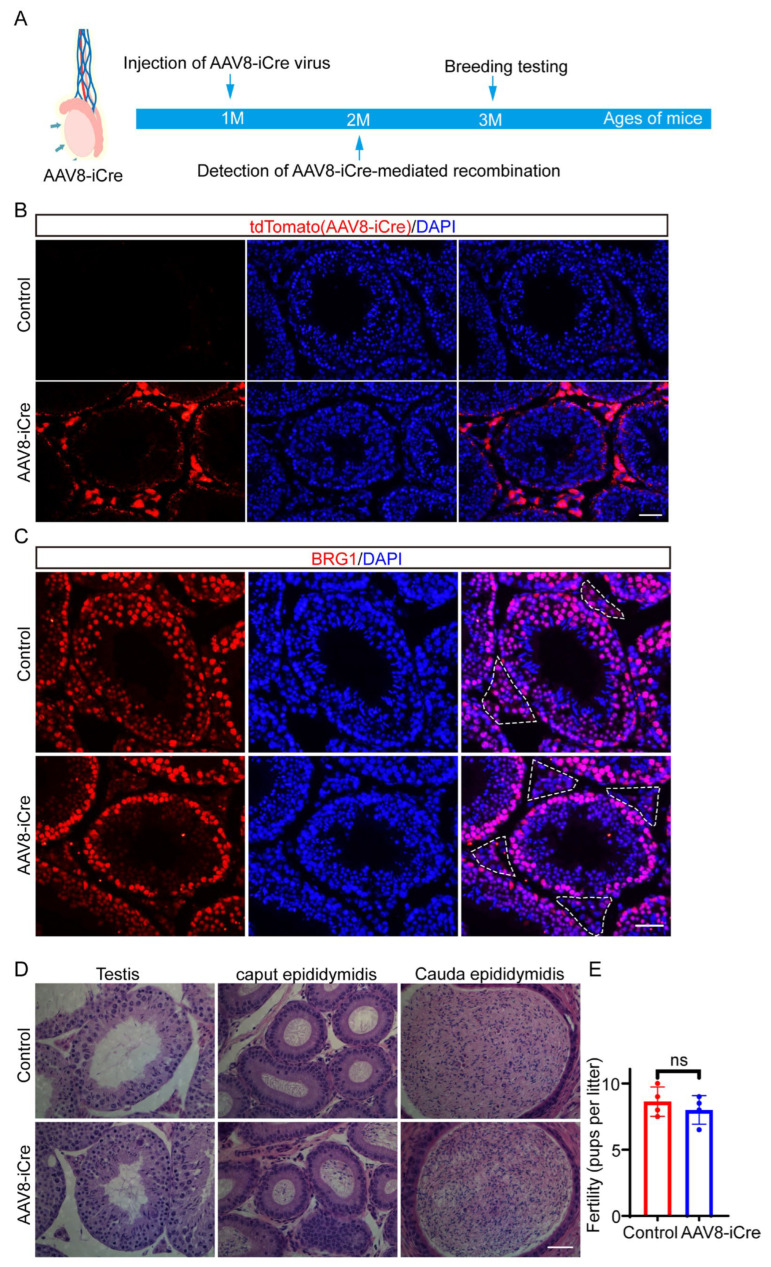
BRG1 deletion in ALCs mediated by AAV8-iCre does not impair male fertility. (**A**) Schematic diagram of intratesticular AAV8-iCre virus injection. Mice were injected at 1 month of age. AAV8-iCre-mediated recombination pattern was assessed at 2 months of age, and fertility was analyzed at 3 months of age. (**B**) Cryosections of testes from *Rosa26-tdTomato* mice injected intratesticularly with AAV8-iCre virus (AAV8-iCre group) or PBS (Control group). tdTomato fluorescence indicates Cre-mediated recombination. (**C**) Testis sections from *Brg1^f/f^* mice injected with AAV8-iCre or control virus, immunostained for BRG1. Dashed line outlines interstitial cells (mainly Leydig cells). (**D**) H&E-stained sections of testes and epididymis from *Brg1^f/f^* mice injected with AAV8-iCre or PBS. (**E**) Quantification of the number of pups per litter. *n* = 4 for each group. Error bars represent SD. ns, not significant, Student’s *t*-tests. Scale bars: 50 μm.

To further corroborate the dispensability of BRG1 in ALCs using an independent approach, we employed an adeno-associated virus (AAV) vector for Cre delivery (Figure 11A). Based on previous studies demonstrating that AAV8 serotype exhibits specific tropism for mouse testicular Leydig cells [34,37], we first validated the specificity and efficiency of the AAV8-iCre vector by intratesticular injection into *Rosa26R-tdTomato* reporter mice. At 30 days post-injection, we detected highly efficient and specific recombination in ALCs mediated by AAV8-iCre (82.12% ± 2.27% of interstitial cells were tdTomato-positive) (Figure 11B).

We subsequently injected AAV8-iCre into the testes of adult *Brg1^flox/flox^* male mice to delete BRG1 in ALCs. Immunofluorescence analysis confirmed efficient BRG1 knockout in the majority of ALCs within the injected testes (76.88% ± 1.11% of interstitial cells were BRG1-negative) (Figure 11C). Consistent with the genetic models, this AAV8-iCre-mediated knockout did not affect testicular morphology (Figure 11D). Histological examination by H&E staining revealed normal seminiferous tubule architecture and ALC morphology, with all stages of spermatogenic cells present and mature sperm abundantly observed in the epididymis, comparable to control injections (Figure 11D). Crucially, mating experiments demonstrated that the fertility of AAV8-iCre-injected males was uncompromised, with litter sizes indistinguishable from those of controls (Figure 11E).

In summary, targeted deletion of BRG1 in ALCs via AAV8-iCre recapitulates the findings from the *PR-Cre* and *Cyp17a1-iCre* models, collectively affirming that BRG1 is not required cell-autonomously in ALCs for their development, spermatogenesis, or male fertility.

## 4. Discussion

In this study, we demonstrate that conditional deletion of BRG1 in ALCs using three independent approaches—*PR-Cre*, *Cyp17a1-iCre*, and AAV8-iCre—does not affect ALC development, spermatogenesis, or male fertility. The dispensability of BRG1 in ALCs is particularly striking given its critical roles in other testicular cell types. In the male germline, BRG1 is required for meiotic progression and DNA repair, and its deletion leads to spermatogenic arrest and infertility [19,20]. By contrast, Sertoli cell-specific deletion of BRG1 does not impair testicular development or spermatogenesis [21], suggesting that BRG1 function is dispensable in some testicular somatic lineages but essential in others. Our findings extend this paradigm to ALCs, revealing that the functional requirement for BRG1 is cell type-specific even within the same tissue.

Several potential mechanisms may explain this functional redundancy. First, the closely related paralog BRM (SMARCA2), which shares high sequence homology and functional similarity with BRG1, may compensate for BRG1 loss in ALCs [38]. In certain cell types, BRM is known to substitute for BRG1 in maintaining SWI/SNF complex function [39,40,41,42]. A previous study reported that BRM is expressed in Sertoli cells but is undetectable in ALCs under basal conditions [21]. Nevertheless, it is possible that BRM expression is upregulated in ALCs following BRG1 ablation. Whether such upregulation occurs in BRG1-deficient ALCs warrants further investigation. Alternatively, it is possible that BRG1-associated BAF complexes are not the primary chromatin remodelers required for ALC differentiation, development, or steroidogenic function. Instead, other chromatin remodeling complexes, such as those from the Imitation Switch (ISWI) or chromodomain helicase DNA-binding (CHD) families, may play the predominant role in orchestrating the chromatin dynamics necessary for ALC lineage establishment, maturation, and steroidogenic gene expression.

Beyond ALCs, our data suggest that BRG1 is likely dispensable for the maintenance or function of peritubular myoid cells and elongated spermatids. In the testis, *PR-Cre* drives robust recombination in peritubular myoid cells and elongated spermatids, implying efficient *Brg1* deletion in these cell types in *PR-Brg1^f/f^* mice. Despite this deletion, we observed no detectable abnormalities in testicular morphology or overall fertility. Notably, we detected little to no BRG1 signal in peritubular myoid cells and elongated spermatids by immunofluorescence, indicating that BRG1 expression is extremely low or absent in these cells under basal conditions. Although this precludes direct assessment of BRG1 deletion efficiency by immunostaining, the lack of detectable BRG1 expression itself supports the notion that BRG1 is unlikely to play a major role in these cell types. Nevertheless, we cannot rule out the possibility that BRG1 deletion causes subtle abnormalities that do not overtly affect testicular morphology or fertility.

A major methodological contribution of this study is the comprehensive characterization of *PR-Cre* expression in the testis, which has not been previously reported. Our findings establish *PR-Cre* as a versatile tool for gene targeting in multiple testicular cell types, with distinct advantages over existing Cre lines. While *PR-Cre* is not exclusively restricted to a single cell type, its expression in other testicular cell populations is limited and well-defined, allowing for informed interpretation of experimental outcomes. We demonstrate that *PR-Cre* efficiently drives recombination in ALCs and their spindle-shaped peritubular progenitors during postnatal development. Several Cre lines have been developed for targeting Leydig cells, including *Amhr2-Cre* [43], *Sf1-Cre* [44], *Cyp17a1-iCre* [22], *Cyp11a1-iCre* [45], *Insl3-iCre* [46], and *Inha-iCre* [47] mice. While these lines have provided important insights into Leydig cell biology, they present certain limitations. Some, such as *Cyp17a1-iCre* [22], *Cyp11a1-iCre* [45], *Insl3-iCre* [46], are predominantly active in differentiated ALCs, with minimal activity in stem/progenitor ALCs, limiting their utility for studying early differentiation events. Others, like *Amhr2-Cre* [43], *Sf1-Cre* [44], and *Inha-iCre* [47], exhibit broad and/or early expression in multiple testicular tissues, which may confound lineage-specific analyses and introduce early developmental defects. In contrast, *PR-Cre* offers the distinct advantage of labeling stem/progenitor ALCs during postnatal development, in addition to efficiently targeting ALCs. This feature may be particularly valuable for studying early ALC differentiation and the establishment of the ALC population.

We also observed robust *PR-Cre* activity in peritubular myoid cells, a cell type that plays an essential role in maintaining seminiferous tubule structure, regulating spermatogonial stem cell niche, and contributing to testicular contractility [48,49]. While existing Cre lines for peritubular myoid cells, such as *smMHC-Cre* (also known as *Myh11-Cre*) [50] and *Sm22a-Cre* [51], have been available, they are not exclusively restricted to the testis and exhibit widespread activity in vascular smooth muscle cells throughout the body. Consequently, *Sm22a-Cre*-mediated deletion of BRG1 results in embryonic lethality [52], and *smMHC-Cre*-mediated deletion causes perinatal lethality in a subset of mice, with surviving animals exhibiting a predisposition to premature death [53]. In stark contrast, *PR-Cre*-mediated BRG1 deletion yields viable and fertile mice with no overt abnormalities, consistent with the absence of reported *PR-Cre* activity in systemic smooth muscle [26]. This phenotypic difference provides compelling indirect evidence that *PR-Cre* does not drive recombination in systemic smooth muscle cells. Thus, *PR-Cre* offers a more focused tool for studying gene function specifically in testicular peritubular myoid cells, as it avoids the confounding effects and potential lethality associated with systemic smooth muscle deletion seen with other Cre lines.

*PR-Cre* also exhibits robust recombination specifically in elongated spermatids, with little to no activity in earlier germ cell stages (spermatogonia, spermatocytes, or round spermatids). The pattern—specific recombination in elongated spermatids with minimal earlier activity—provides an additional valuable tool for studying genes required during the final stages of spermiogenesis, a critical period for sperm morphogenesis, flagellum formation, and nuclear condensation. Several Cre lines targeting elongated spermatids have been previously described [54,55,56]; *PR-Cre* thus expands the available toolkit for investigating this important stage of spermatogenesis.

We also detected limited ectopic *Cyp17a1-iCre* activity in a subset of Sertoli cells and peritubular myoid cells, a finding not previously reported. This observation warrants caution when interpreting phenotypes in studies using this Cre line, as even low-level ectopic recombination could potentially confound lineage-specific conclusions. Furthermore, the detection of this ectopic activity was enabled by the sensitive tdTomato reporter, suggesting that fluorescent reporters may offer greater sensitivity than traditional β-galactosidase-based methods for characterizing Cre activity patterns. Reassessment of existing Cre lines using more sensitive detection approaches may therefore reveal previously unrecognized expression patterns.

While *PR-Cre* efficiently recombines the *Rosa26* reporter locus in most principal cells, BRG1 deletion in the epididymis was unexpectedly observed in only a minor fraction of these cells. This striking discrepancy highlights the critical caveat that reporter activity may overestimate functional knockout efficiency due to locus-specific differences in recombination threshold and chromatin accessibility. Of note, in most other cell types examined—including ALCs (for *PR-Cre*, *Cyp17a1-iCre*, and AAV8-iCre) and epididymal principal cells using *Cyp17a1-iCre*—the tdTomato reporter pattern correlated well with actual BRG1 deletion efficiency, suggesting that reporter activity can largely reflect target-gene deletion in many contexts. Nevertheless, the discordant observation in the *PR-Cre* epididymis underscore the necessity of directly validating target gene deletion, as reporter activity alone may not accurately reflect functional knockout efficiency.

Collectively, this study demonstrates that BRG1 is not required for ALC development, differentiation, or function, as revealed by three independent conditional deletion strategies. Furthermore, BRG1 is also dispensable in peritubular myoid cells, elongated spermatids, and epididymal principal cells. Additionally, we provide the first systematic characterization of *PR-Cre* expression in the male reproductive system, establishing it as a versatile tool for targeting stem/progenitor ALCs, peritubular myoid cells, and elongated spermatids. This work thus offers both functional insight into chromatin regulation in testicular cells and a valuable methodological resource for future studies in male reproductive biology.

## 5. Conclusions

In summary, this study provides systematic characterization of *PR-Cre* activity in the male reproductive system. We demonstrate that *PR-Cre* drives recombination in multiple testicular cell types, including stem/progenitor ALCs, peritubular myoid cells, and elongated spermatids, establishing it as a versatile new tool for male reproductive research. Using three independent conditional deletion approaches (*PR-Cre*, *Cyp17a1-iCre*, and AAV8-iCre), we further show that BRG1 is dispensable for ALC development, steroidogenic function, and male fertility. These findings extend our understanding of the cell type-specific requirements for SWI/SNF chromatin remodeling complexes within the testis and provide a valuable methodological resource for future Cre-loxP-based genetic studies.

## Data Availability

The original contributions presented in this study are included in the article/Supplementary Materials. Further inquiries can be directed to the corresponding author.

## Supplementary Materials

The following supporting information can be downloaded at: https://www.mdpi.com/article/10.3390/biom16060816/s1, Table S1: Sequences of primers used for the real-time PCR.
