# Supplementary material for: Characterization of PR-Cre Activity in the Testis and Its Application Reveals BRG1 Is Dispensable in Adult Leydig Cells
Source: Biomolecules. 2026 May 31;16(6):816. doi: 10.3390/biom16060816 (PMC13297161; doi:10.3390/biom16060816)
Supplement: Supplementary file 1 [file biomolecules-16-00816-s001.zip › biomolecules-4313074-supplementary.pdf]

Supplementary Materials

Table S1. Sequences of primers used for the real-time PCR.

Target Gene	Sequence (5′ to 3′)	Direction
Star	ATGTTCCTCGCTACGTTCAAG	Forward
	CCCAGTGCTCTCCAGTTGAG	Reverse
Cyp11a1	AGGTCCTTCAATGAGATCCCTT	Forward
	TCCCTGTAAATGGGGCCATAC	Reverse
Cyp17a1	GCCCAAGTCAAAGACACCTAAT	Forward
	GTACCCAGGCGAAGAGAATAGA	Reverse
Hsd3b1	TGGACAAAGTATTCCGACCAGA	Forward
	GGCACACTTGCTTGAACACAG	Reverse
Hsd17b3	AGGTTCTCGCAGCACCTTTTT	Forward
	CATCGCCTGCTCCGGTAATC	Reverse
β-actin	GTGACGTTGACATCCGTAAAGA	Forward
	GCCGGACTCATCGTACTCC	Reverse
